# P132: Hand hygiene “before aseptic tasks”: a critical point even at a hematology and transplant ward

**DOI:** 10.1186/2047-2994-2-S1-P132

**Published:** 2013-06-20

**Authors:** S Scheithauer, B Batzer, C Pino Molina, A Widmer

**Affiliations:** 1 Infection Control, University Hospital Basel, Basel, Switzerland; 2 Infection Control & Infectious Diseases, University Hospital Aachen; RWTH Aachen, Aachen, Germany; 3 Hematology, University Hospital Basel, Basel, Switzerland

## Introduction

A high compliance with hand hygiene especially before aseptic tasks (indication 2) seems to be of greatest importance in order to minimize hospital acquired infections and bacterial cross transmission.

However, the majority of indication-specific analyses revealed the lowest compliance with indication 2.

## Objectives

In order to evaluate hand hygiene events (HHE) “before aseptic tasks” we correlated HHE at the patients` bedside and at the laminar air flow benches with the number of invasive procedures performed in the patient room and at the benches, respectively.

## Methods

Real time and location-specific assessment of all HHE occuring at a hematology ward (University Hospital Basel, Switzerland) was performed from 01.03.12 to 28.02.13 using the Ingo-man Weco; Ophardt Hygienetechnik, Issum; Germany. The total number of HHE as well as all HHEs performed in the patient rooms and at the benches were correlated with the number of invasive procedures performed in the patients rooms (change of wound dressing; application of i.v. medications, blood withdrawel/puncture of vessels, lumbar/bone marrow puncture) and at the benches (preparing i.v. formulations) on a daily basis. Data on invasive procedures derived from the electronic patient data sheet.

## Results

A total of 208.184 HHE occurred, thereof 17.311 (8%) in the patient rooms. These figures translate into 57 HHE/ patient-day (PD) in general and to 5 HHE in the patient rooms/PD. However, a total of 65.981 invasive procedures (18/PD) were performed in the patient rooms. Calculating (at least) two HHEs for an invasive procedure at the patient side (indication 2 and 3) compliance was only 14% (5/36) for these indications. At the laminar air flow benches a total of 32.605 HHE (16%) were performed, translating into 9 HHE/PD. The number of aseptic tasks (indication 2 only) performed at the benches added up to 28.418 (8/PD). Thus, compliance while preparing i.v. medications reached 87%.

## Conclusion

Compliance before aseptic tasks at the patient room seemed to be unacceptable low even at wards caring for high risk patients. Nevertheless, compliance for i.v. preparations performed at the laminar air flow seemed to be high. The latter may be explained by the great importance attached to these procedures.

## Disclosure of interest

None declared

